# An Integrated Transcriptome and Proteome Analysis Reveals New Insights into Russeting of Bagging and Non-Bagging “Golden Delicious” Apple

**DOI:** 10.3390/ijms20184462

**Published:** 2019-09-10

**Authors:** Gaopeng Yuan, Shuxun Bian, Xiaolei Han, Shanshan He, Kai Liu, Caixia Zhang, Peihua Cong

**Affiliations:** 1 Key Laboratory of Biology and Genetic Improvement of Horticultural Crops, Xingcheng 125100, China; 2 Research Institute of Pomology, Chinese Academy of Agricultural Sciences, Xingcheng 125100, China

**Keywords:** fruit russeting formation, transcriptome, proteome, bagging and non-bagging, “Golden Delicious” apple, lignin biosynthesis

## Abstract

Apple skin russeting naturally occurs in many varieties, particularly in “Golden Delicious” and its pedigree, and is regarded as a non-invasive physiological disorder partly caused by excessive deposition of lignin. However, the understanding of its molecular mechanism is still limited. In this study, we used iTRAQ (isobaric tags for relative and absolute quantitation) and RNA-seq to detect the changes in the expression levels of genes and proteins in three developmental stages of russeting formation, in russeted (non-bagging) and non-russeted (bagging) skin of “Golden Delicious” apple. 2856 differentially expressed genes and 942 differentially expressed proteins in the comparison groups were detected at the transcript level and protein level, respectively. A correlation analysis of the transcriptomics and proteomics data revealed that four genes (MD03G1059200, MD08G1009200, MD17G1092400, and MD17G1225100) involved in lignin biosynthesis are significant changed during apple russeting formation. Additionally, 92 transcription factors, including 4 LIM transcription factors, may be involved in apple russeting formation. Among them, one LIM transcription factor (MD15G1068200) was capable of binding to the PAL-box like (CCACTTGAGTAC) element, which indicated it was potentially involved in lignin biosynthesis. This study will provide further views on the molecular mechanisms controlling apple russeting formation.

## 1. Introduction

“Golden Delicious” is an economically important cultivar in the world, and plays a core role in apple genetic breeding [1]. However, it presents a phenotype of russeted skin (known as fruit russeting), and its pedigree also display the similar phenotype more or less. Fruit russeting also appears in waxy apples and causes a russeted surface, which is typically rough and corky, as well as a reduced storage life [2]. Fruit russeting is generally considered as an undesirable trait by consumers and millions of dollars are lost annually [2,3].

Fruit russeting is closely related to lacks and defects of the cuticular and epidermal layers [4]. The outbreak of apple russeting results from the damage of epidermal cells that occurs within the 30 to 60 days after flowering (DAF), when is the most sensitive period to external environment for apple [5]. During the period, apples have a sigmoid growth pattern and the epidermal cells are undergoing the most rapid rate of cell division and enlargement. At the same time, cuticle growth speed is slower than epidermal layers, without synchronous growth, resulting in the formation of microcracks [5]. Later, the epidermal cells burst and the underlying cork cambium accumulates rapidly to form brown particles, which becomes visible as the familiar “russeting” [2,4] and comes into being the typical brown and corky phenotype of “russeted” apples [6].

A wide range of external factors that impact russeting formation heavily have been identified, such as surface moisture and humidity [7], temperature [2], mechanical wounding [8], infection by pests or microorganisms [9], or the application of plant protection agents [10]. To date, few treatments are able to effectively limit russeting occurrence, including utilization of chemical agent, spraying fungicides, and fruit bagging [11,12]. For example, the application of gibberellic acid (GA_4+7_) can lead to reduction of the cuticle microcracking of “Golden Delicious” due to decreased surface tension of epidermal cell, resulting in a significant decrease of the russeting phenotype [3,5]. However, the application of phytohormones will bring some disadvantageous effects on to the plant, such as the tillering capacity of branches as well as the flower and yield in the next year [13]. Bagging will lead to an increase in titratable acid content and a decrease in sugar content though it is another effective measure to prevent apples from russeting [14,15]. Despite the physical and chemical methods mentioned above can inhibit the occurrence of apple russeting to a certain extent, they waste a lot of resources and pollute the environment. Therefore, in order to breed new non-russeted apple varieties and understand the genetics and potential mechanism of apple russeting formation, breeders have carried out extensive breeding research [16]. But little progress has been achieved in these areas, mainly because of the long breeding cycle of apples.

Despite tangible progress in the aetiology and in phenology of apple russeting, the underlying genetics is still poorly understood. A theory that the trait was under genetic control was first presented by Falginella’s team [17]. They found that some russeted apples derived from mutation of non-russeted cultivars and the offspring of controlled hybrids had a certain separation ratio of the character [2,18]. Based on segregation (1:1) observed in the progeny of “Court Pendu Plat” and “D’Arcy Spice”, Alston indicated that complete russeting might be controlled by a simple gene (Ru gene) [19]. Conversely, non-complete russeting controlled by multi-factors had been reported in references, with the evaluation of polygenic from combinations between partially russeted and either slightly to full russeted varieties [19]. According to a separation ratio of moderate russeted cultivars “Cox’s Orange Pippin” hybrid offspring, Alston and Watkins stated that a major gene played a leading role and was modulated by further minor genes [19]. In recent years, studies on the molecular level of apple russeting have gained some progress. In Malus × domestica, a previous study indicated that the MdSHN3 transcription factor prevented apples from russeting due to its overexpression and increased cuticle deposition [16]. Another study showed that the MdMYB93 transcription factor implicated in apple russeting was related to suberin synthesis and regulation [20]. When MdMYB93 gene was further transiently overexpressed in Nicotiana benthamiana leaves, the key related-genes in suberin biosynthetic pathway were up-regulated and the content of suberin also increased, indicated that MdMYB93 transcription factor promoted the formation of apple russeting [21]. Later, it was proved that MdMYB53 transcription factor possessed a similar function as MdMYB93, which was a MYB107 and MYB9 homolog regulating suberin deposition in apple [22]. Most studies on apple russeting mostly focused on transcription factors or genes involved in the biosynthesis of cuticle or suberin, and they are also involved in phenylpropanoid or lignin biosynthesis. And it had been reported that several genes or transcription factors related to phenylpropanoid biosynthesis. For example, in “Dangshansuli” pear russeted mutant “Xiusu”, genes regulating enzymes involved in lignin biosynthesis were up-regulated, such as PAL (phenylalanine ammonia-lyase), CCR (cinnamyl CoA reductase), CAD (cinnamyl-alcohol dehydrogenase) and POD/PRX (peroxidase), indicating those genes had a positive correlation with the russeting formation [23]. Besides, study on Malus × domestica found that apple russeting may be closely related to higher accumulation of phloridzin in the skin, and the synthetic precursors of phloridzin were regulated by PAL, C4H (cinnamate hydroxylase), and 4CL (4-coumaric acid- CoA ligase). It suggested that additional transcription regulators or genes related to phenylpropanoid or lignin biosynthesis may play important roles in the formation of apple russeting [24].

In spite of the great achievements, further efforts are still needed to enrich the regulatory mechanism of apple russeting. And although more candidate proteins or genes can be obtained through proteomics and transcriptomics, it will inevitably lead to the appearance of pseudo-positive genes, because gene expression is not only a one-way flow from transcriptome to proteome, but a connection between them. In the current study, russeted and non-russeted “Golden Delicious” apples were used as the experimental material for scanning electron microscopy (SEM), light microscopy (LM), RNA sequencing, and protein quantification, as well as RNA and protein correlation analysis at three developmental stages (DAF 30, DAF 60, and DAF 150). Candidate genes and transcription factors involved in various aspects of apple russeting were identified, including phenylpropanoid and lignin biosynthesis, such as MD17G1092400, MD08G1009200, MD17G1225100, MD03G1059200, and MD15G1051000, MD15G1068200, etc. Meanwhile, a LIM transcription factor was identified to regulate the expression of genes related to lignin biosynthesis. The study has important significance for further breeding of non-russeted apple. 

## 2. Results

### 2.1. Histological Analysis of Russeting Apple

In the present study, we found that bagged fruits were russeting-free, and non-bagged fruits showed heavy russeted skin, which formed on the stem hollow, stalk, and top of apple body (Supplementary Figure S1). The formation and development of fruit russeting were affected by the developmental stages of apple. At 30 days after flowering (DAF), small russeting-like particles formed on the surface of apple (Supplementary Figure S2A), then the particles joined together as a whole at DAF60 and formed brown spots (Supplementary Figure S2B). As time went by, spots protruded from the pericarp at DAF150 (Supplementary Figure S2C) and affected the fruit quality seriously.

**Figure 1:**
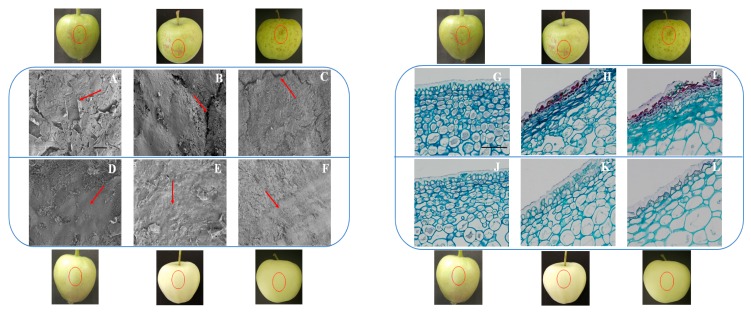
The structure and epidermal cells changes of the russeted and non-russeted fruit skin of “Golden Delicious” apple: (**A**) waxy layer microcracks and warped at 30 days after flowering (DAF30) (CK1); (**B**) “V”-shaped crack at DAF60 (CK2); (**C**) “V”-shaped crack became much longer and deeper at DAF150 (CK3); (**D**) waxy layer at DAF30 (T1); (**E**) waxy layer at DAF60 (T2); (**F**) waxy layer microcracks at DAF150 (T3); (**G**) waxy layer and epidermal cells at DAF30 (CK1); (**H**) the waxy layer broken, and first two epidermal cells ruptured with russeted compositions depositing (CK2); (**I**) the ruptured epidermal cells nearly to 10 layers at DAF150 (CK3); (**J**) waxy layer and epidermal cells at DAF30 (T1); (**K**) waxy layer became thicker and epidermal cells became larger at DAF60 (T2); (**L**) waxy layer became much thicker and epidermal cells became much larger at DAF150 (T3). A, B, C, G, H, I belonged to CK group, and D, E, F, J, K, L belonged to T group. Scale length was 50 μm.

For further analyses of the structural changes of the russeted and non-russeted fruit skin, we carried out a SEM experiment (Figure 1). The waxy layer of the epidermis of russeted fruit became warped with microcracks at DAF30 (Figure 1A), and then the microcracks became bigger and showed a “V-shaped” crack at DAF60 (Figure 1B). However, the skin of T group (bagging group) smooth without microcracks at both stages (Figure 1D,E). At DAF150, the “V-shaped” crack became much longer and deeper, while microcracks appeared in T group without obvious changes (Figure 1C,F).

Afterwards, we assessed the changes of epidermal cells through the LM experiment (Figure 1). Rupture degree of epidermal cells of CK group (non-bagging group) became larger during different developmental stages. At DAF60 (Figure 1H), the russeted compositions began to deposit and showed purple color, and the epidermal cells in the first two layers appearing irregularly arrangement due to compression showed minor rupture. At DAF150 (Figure 1I), the degree of rupture of cells gradually deepened, the number of irregularly arranged cells increased to nearly 10 layers, with the russeted compositions deposited heavily. However, there were no significant changes in T group during the three stages (Figure 1J–L). 

We also analyzed the thickness of the wax layer and the size of the epidermal cells, which showed that the thickness of the wax layer increased with developmental stages. While the thickness of T group was all significantly greater than that of CK group among the three stages (Supplementary Figure S3A). The size of epidermal cells also showed a similar trend in the wax layer structure (Supplementary Figure S3B), increasing by 46% and 73%, respectively.

### 2.2. General Characterization of Transcriptome Data

To obtain key genes involved in the formation of the russeting process in apples. RNA-seq was used to determine genes linked to a trait of interest. For this purpose, 18 cDNA libraries were built from mRNA extracted from the skin of “Golden Delicious” apple with russeted (non-bagged) and non-russeted (bagging) phenotype. As a result, the average output data of samples was 10.78 Gb, the match percent of samples to reference genomes was 91.59%, and that of gene sets was 86.26% (Supplementary Table S1). In this study, a total of 40,781 genes were identified, including 38,878 known genes and 1903 predicted new genes. A total of 22,110 new transcripts were detected and used in Mascot search database for MS protein identification. Among them, 16,756 belonged to the new variable splicing subtype of known protein-coding genes, 1949 belonged to the transcripts of new protein-coding genes, and the remaining 3405 belonged to long-chain non-coding RNA. Reads coverage over 80% accounts for more than 63% of the total number of transcripts.

**Figure 2:**
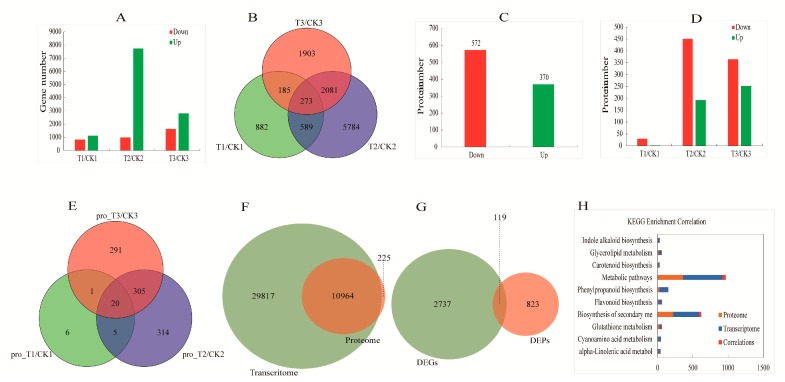
Analysis of differentially expressed genes (DEGs), differentially expressed proteins (DEPs) and correlations of comparative groups of “Golden Delicious” apple skin. (**A**) the statistics of DEGs of T1/CK1, T2/CK2, T3/CK3; and (**B**) venn diagram of DEGs in various comparative groups; (**C**) the statistics of DEPs of pro_T/CK; (**D**) the statistics of DEPs of pro_T1/CK1, pro_T2/CK2, pro_T3/CK3; and (**E**) venn diagram of DEGs in various comparative groups; (**F**) the venn diagram of the number of associations between proteome and transcriptome at the identification level; (**G**) the venn diagram of the number between DEGs and DEPs at difference level; (**H**) KEGG (Kyoto Encyclopedia of Genes and Genomes) pathway enrichment analysis of 52 DEGs and DEPs.

There were total 2856 DEGs in the comparison groups of T1/CK1, T2/CK2, and T3/CK3, the number of up-regulated genes was ~3.4 times more than the number of down-regulated genes, the number of DEGs increased and then decreased following russeting formation (Figure 2A). Totally 273 DEGs were screened in a Venn diagram among the three stages between russeted and non-russeted apple skin (Figure 2B).

**Figure 3:**
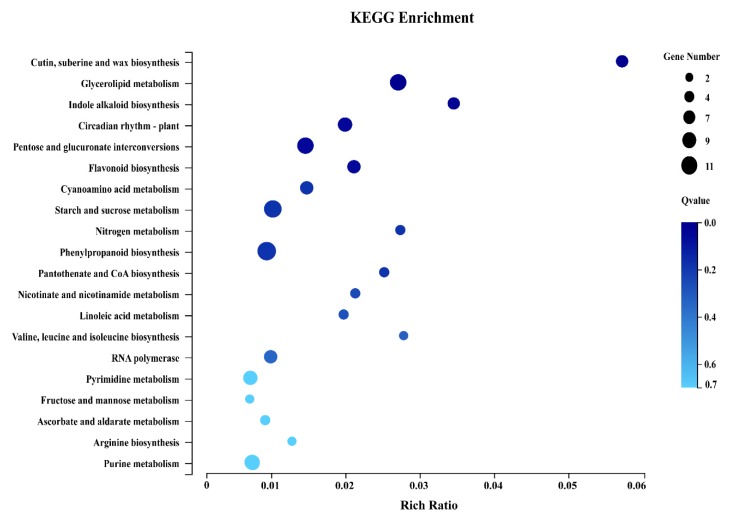
Bubble diagram of enrichment of KEGG pathway. *X* axis for the enrichment Ratio (selected genes concentrated annotation to a particular item number comments to this entry with this species the Ratio of the total number of genes, calculating formula for Rich thewire = Term Candidate Gene Num/Term Gene Num). *Y* axis to KEGG Pathway, the size of the bubbles represented annotation to a KEGG Pathway on the number of genes, color represents enrichment Qvalue value, the deeper the color represents the Qvalue value is smaller.

To further determine which biological pathways was significantly different during russeting development (FDR ≤ 0.01), KEGG pathway enrichment analysis was performed for the DEGs (Figure 3). The most enriched categories were the phenylpropanoid biosynthesis, starch and sucrose metabolism, and glycerolipid metabolism. In addition, the cutin, suberine, and wax biosynthesis, as the key category mentioned in previous study [20], was also annotated in our work. There were totally 11 genes in phenylpropanoid biosynthesis pathway, and most of them were related enzymes to lignin biosynthesis (Table 1), such as CAD (cinnamyl-alcohol dehydrogenase, MD01G1042500 and MD01G1042800), PDC (pyruvate decarboxylase, MD03G1126700), HCT (shikimate O-hydroxycinnamoyl transferase, MD17G1157600) and PDA (phospholipid: diacylglycerol acyltransferase, MD02G1161000, MD11G1153800, MD11G1214800, and MD12G1252500). In cutin, suberine and wax biosynthesis (Table 2), all of the 5 genes were related enzymes of suberin, such as CYP86A1 (long-chain fatty acid omega-monooxygenase, MD03G1073600), CYP86B1 (fatty acid omega-hydroxylase, MD13G1004700), and HTT1 (omega-hydroxypalmitate O-feruloyl transferase, MD08G1058900, MD09G1007600, and MD17G1011500).

### 2.3. General Characterization of Proteome Data

A total of 2,640,114 spectra were generated from the 18 tested samples, and 54,338 peptides and 11,189 plant proteins were identified (Supplementary Table S2). 

There were 942 differentially expressed proteins (DEPs) identified between russeted and non-russeted apple skin (Figure 2C), and the number of down-regulated DEPs was ~1.5 times more than up-regulated DEPs. To explore the differences in biological pathways of the DEPs in detail, KEGG pathway enrichment analysis was conducted, and there were 834 of 942 DEPs annotated pathways (Supplementary Table S3). In the top 20 pathways, the most enriched category was the metabolic pathway which had 362 DEPs. Interestingly, the pathway of phenylpropanoid biosynthesis (28 DEPs) that we are concerned about was also included into the 20 pathways, and the up-regulated proteins of it were equal to the down-regulated proteins.

When comparing the number of DEPs at the three stages during the formation of apple russeting, we found that the highest number of DEPs was shared at DAF60 stage (644), followed by the DAF150 stage (617) and the DAF30 stage (32) (Figure 2D). There were only 20 DEPs of Venn diagram among the three stages and most of them were protein enzymes (Figure 2E), including 5 enzymes involved in the phenylpropanoid biosynthesis (MD05G1122100, MD05G1209300, MD08G1168600, MD10G1195700, and MD15G1222600) (Table 3).

### 2.4. Proteome and Transcriptome Correlation Analysis

To further understand the relationship between proteome data and transcriptome data, a research about correlation analysis was carried out. The parameters used for correlative analysis and the number of proteins and genes in the correlative analysis are listed in (Supplementary Table S4).

The results showed that 10,964 genes and proteins (Figure 2F) and 119 DEGs and DEPs (Figure 2G) were correlated in the transcriptome and proteome of different treatments, respectively, and a correlation using Spearman correlation test (R = 0.36, Supplementary Figure S4). According to the cluster analysis, which was performed on the 119 DEPs and DEGs, we found that 110 of them showed a same trend, and the other 9 DEPs showed an opposite trend. Among the 110 proteins and genes with the same trend, 15 of them were up-regulated and 95 were down-regulated (Supplementary Figure S5, and Supplementary Table S5). To better understand the function of these 119 DEPs and DEGs, we performed a KEGG enrichment analysis, and the results showed that 52 genes/proteins were annotated into 10 KEGG pathways (Figure 2H), including the metabolic pathways, biosynthesis of secondary metabolities, phenylpropanoid biosynthesis pathway, etc. There were 5 DEPs and DEGs in the phenylpropanoid biosynthesis, including 3 down-regulated genes, such as E3 ubiquitin-protein ligase UBR (UBR4, MD00G1116600), peroxidase (PRX, MD17G1092400), cinnamyl-alcohol dehydrogenase (CAD9, MD08G1009200), and 2 up-regulated genes, such as shikimate O-hydroxy cinnamoyl transferase (HCT, MD17G1225100), peroxidase (PRX, MD03G1059200) (Table 4). Interestingly, except UBR4, the other 4 DEGs and DEPs may be directly involved in lignin biosynthesis. The result indicated that lignin related gene may affect the formation of apple russeting.

### 2.5. Identification of Transcription Factors Involved in Lignin Biosynthesis

**Figure 4:**
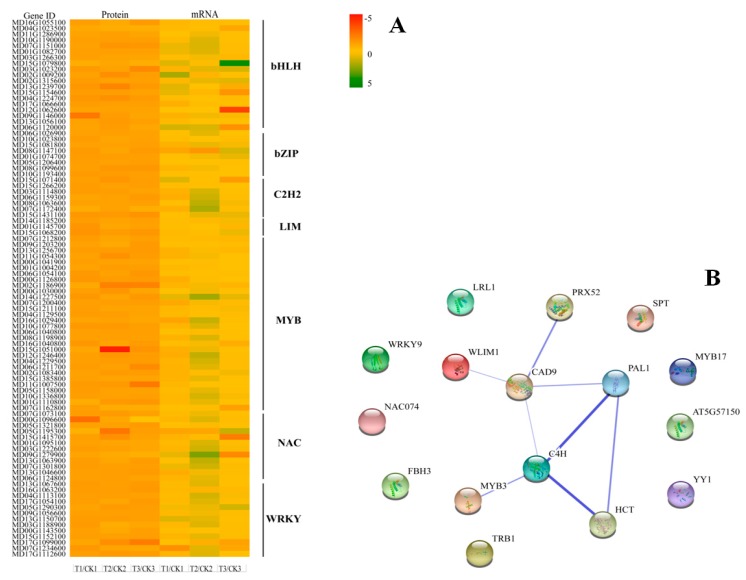
Identification of transcription factors (TFs) involved in lignin biosynthesis. (**A**) Analysis of identified TFs during fruit russeting formation and development of “Golden Delicious” apple. The relative changes in abundance of mRNA are shown using a log2 scale. (**B**) Lignin candidate genes interaction matrix performed in STRING using the TAIR accessions of PRX52, MD03G1059200; PAL1, MD04G1096200; CAD9, MD08G1009200; HCT, MD17G1225100; C4H, MD03G1051400; GATA type zinc finger transcription factor family protein (WLIM1, MD15G1068200); MYB-type transcription factor (MYB3, MD15G1051000; TRB1, MD02G1186900; MYB17, MD11G1007500); WRKY family transcription factor (WRKY9, MD17G1099000); Basic helix-loop-helix (bHLH) DNA-binding superfamily protein (AT5G57150, MD13G1239700; SPT, MD09G1146000; LRL1, MD04G1224700; FBH3, MD03G1023200); NAC domain containing protein (NAC074, MD05G1195300); Zinc finger (C2H2 type) family protein (YY1, MD15G1431100). Blue lines represent the gene interaction confidence (ranged from 0 to 1); thickest lines display confidence scores higher than 0.9, thinnest lines display confidence scores between 0.3 and 0.8.

In order to identify the transcription factors (TFs) involved in lignin biosynthesis of apple russeting, we conducted a targeted analysis of TFs from all identified genes and proteins. Totally 92 TFs (19 bHLH, 8 bZIP, 7 C2H2, 4 LIM, 18 MYB, 23 NAC, and 13 WRKY) were screened during the formation and development of fruit russeting (Figure 4A), among which there were 11 differentially abundant TFs, they were all up-regulated (Supplementary Table S6). 

According to the KEGG pathway, *HCT*, *PRX,* and *CAD9* may be directly involved in the regulation of lignin biosynthesis. In order to further search for the regulatory factors of these genes, we built a functional protein association network by using differentially abundant TFs and genes that mentioned above (Supplementary Table S6). The results showed that WLIM1 and MYB3 participated in the regulation of lignin biosynthesis related-genes, interestingly, WLIM1 acted directly on CAD9 (Figure 4B), which may catalyze the final step specific for the production of lignin monomers (KEGG pathway, ko00940). The results indicated that LIM TF may play a key role in the formation of apple russeting.

### 2.6. Quantitative Real-Time PCR Confirmation of Selected Genes

**Figure 5:**
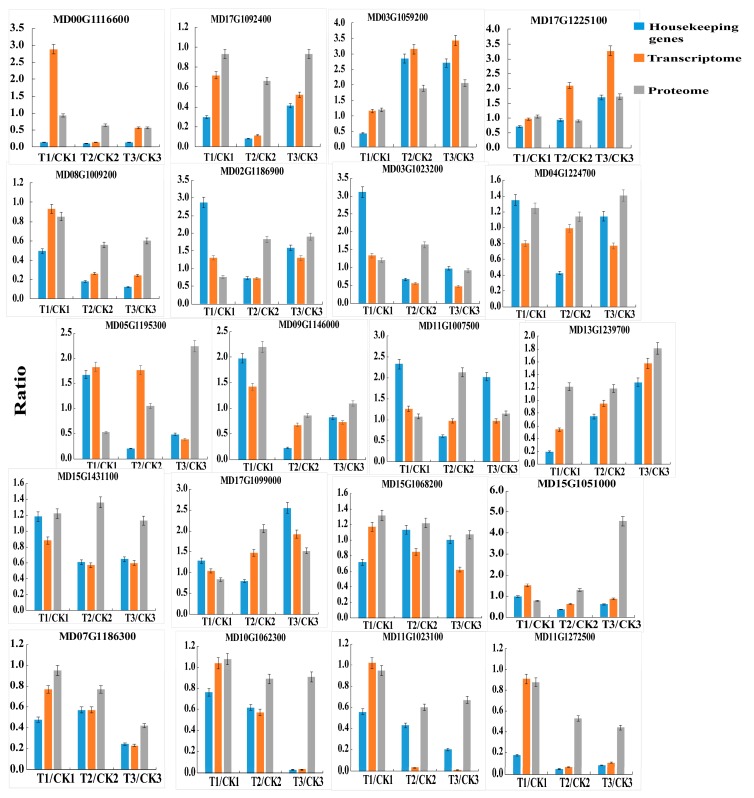
qRT-PCR validation of differential gene expression. The ratio of transcript and protein profiles were calculated by RNA-Seq and iTRAQ, respectively.

To validate the expression level of transcriptome and proteome in this study, 20 genes, including 5 genes of Table 4, 11 TF-encoding genes of Figure 4B, and 4 genes involved in suberin biosynthesis (MD07G1186300, MD10G1062300, MD11G1023100, and MD11G1272500) were selected for qRT-PCR analysis. We used two reference genes to normalize our data, and the expression patterns detected by qRT-PCR for most of these 20 genes were consistent with those in the transcript and protein profiles, which indicated that our mRNA and protein data were reliable (Figure 5).

### 2.7. Functional Identification of MD15G1068200

**Figure 6:**
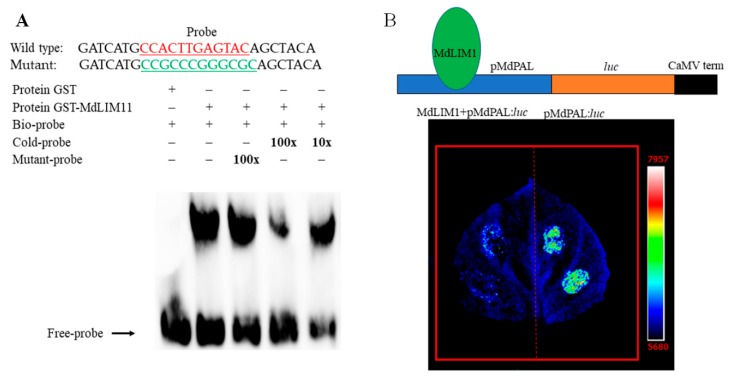
Functional Identification of MdLIM1. (**A**) MdLIM1 binds directly to the PAL-box element CCACTTGAGTAC. (**B**) Constructs and transient expression assays showed that MdLIM1 obviously inhibited the luciferase expression levels. Upper panel, the pMdPAL:luc construct backbone consists of the promoter from MdPAL (pMdPAL, blue box), luciferase ORF and cauliflower mosaic virus terminator (orange and black box). Luciferase image of Nicotiana benthamiana leaves 72 h after infiltration with the Agrobacterial strains containing MdLIM1+pMdLIM1:luc (left), and pMdLIM1:luc (right), respectively. The scale range was 5680 to 7957.

Phylogenetic analyses showed that the protein sequences of MdLIM1 and NtLIM1 belong to the same branch (Supplementary Figure S6). Previous study have clearly shown that NtLIM1 regulated the related genes expression of lignin biosynthesis by binding to the PAL-box (CCA(C/A)(A/T)A(A/C)C(C/T)CC) in tobacco [25], so we predicted that MdLIM1 had similar function with NtLIM1. Then we carried out an EMSA test to verify the ability of MdLIM1 to combine PAL-box element, the results showed that MdLIM1 was capable of binding to the PAL-box like (CCACTTGAGTAC) element (Figure 6A), which indicated it was potentially involved in lignin biosynthesis.

To further determine whether MdLIM1 promotes or inhibits the related genes expression of lignin biosynthesis. We performed luciferase reporter assays in *Nicotiana benthamiana* leaves. The results showed that the agrobacterium co-injection of MdLIM1 and *MdPAL* promoter led to a significant decrease in reporter gene expression, relative to the promoter alone, suggesting that MdLIM1 transcription factors modulate the expression of lignin related genes and further affect the synthesis of lignin (Figure 6B).

## 3. Discussion

### 3.1. Histologic Change of Russeted Skin

The studies on apples and pears had proven that bagging can significantly reduce the occurrence of fruit russeting [26]. In this study, we found that apple russeting was reduced extremely after bagging, which might be due to the change of epidermal structure [27,28]. Skin crack was the morphological basis of fruit russeting formation, and the waxy layer and cuticle acted as the outer layers of the apple peel to protect the epidermal structure from breaking down [29]. Through SEM experiment, we observed the cracks of russeted fruit skin became gradually deeper with fruit development, then the structural feature produced powerful conditions for the formation of fruit russeting. The non-russeted fruit skin showed minor microcracks because the bagged fruits had a greater toughness on the surface. We also found that the non-russeted fruit had a thicker waxy layer and larger epidermal cells, and the waxy layer distributed uniformly and the epidermal cells were tightly arranged in a regular pattern when comparing with the russeted fruit.

### 3.2. Changes in Related Genes of Lignin Biosynthesis

Although the morphology of the fruit russeting formation is well understood, the knowledge on the genetic level remains unclear to this moment. Legay et.al [20] revealed abundant cell wall-related DEGs involved in stress response signals and regulatory mechanism in russet and waxy apples, which may lead to the synthesis and deposition of suberin. In current study, there were only 5 genes involved in biosynthesis of suberin, which may be the disadvantage of bagging compared to the comparison between fully-waxy and fully-russeted varieties [20]. However, related studies had shown that fruit russeting was closely related to the excessive accumulation of lignin, which suggested that lignin may play important roles in the formation of fruit russeting [30]. The biosynthesis of lignin involves various enzymes, including PAL, C4H, 4CL, HCT, coumaric acid 3-hydroxylase (C3H), cinnamyl CoA reductase (CCR), CAD, and POD [31]. In pears, some related enzymes activity of lignin biosynthesis was inhibited after bagging, such as PAL, 4CL, C4H, CAD, and POD, resulting in the inhibition of fruit russeting, indicating the related genes expression had changed [32]. In our study, the KEGG enrichment analysis showed that biosynthesis of PAL, HCT, CAD, PRX, and some other enzymes may be involved in lignin biosynthesis pathways, and genes associated with these enzymes were up/down-regulated, suggesting that the transcription levels of these genes had changed after bagging. 

Among these enzymes of the lignin biosynthesis pathway, PAL was regarded as a marker enzyme for the beginning of lignification [33]. In *Arabidopsis thaliana*, 4 *PAL* members were all expressed in the inflorescence stalk with high content of lignified cells [34]. In the *pal* mutant *Arabidopsis thaliana* plant, PAL enzyme activity and lignin content were both reduced significantly [35]. In our transcriptome data, one *PAL* gene (MD04G1096200) was significantly down-regulated, indicating that the biosynthesis of lignin may be inhibited. Also, one down-regulated *HCT* gene was screened in our transcriptome data, and studies have shown that inhibiting the expression of *HCT* gene leads to changes of lignin contents of *Arabidopsis thaliana* and *Nicotiana tabacum*, indicating that *HCT* involved in the biosynthesis and transformation of lignin monomer [36,37]. Besides, In *Arabidopsis thaliana AtCAD4/AtCAD5* mutants, the lignin content of the stem was significantly reduced by 94%, suggesting that *AtCAD4* and *AtCAD5* were related to lignin biosynthesis [38]. Here we found three down-regulated *CAD* genes (MD01G1042500, MD01G1042800 and MD08G1009200) in the data of transcriptome and correlation analysis, estimating that *CAD* may be involved in the formation process of fruit russeting. Furthermore, POD/PRX is indispensable in the process of polymerization lignin monomers into lignin macromolecules [39]. Studies have reported that silencing *PrxA3a* gene leads to lignin reduction in aspen [40], and overexpressing *FaPOD27* obviously increased lignin content in strawberry [41,42]. We found two *PRX* genes in the data of correlation analysis, indicating that they may play important roles in the synthesis of lignin during the formation of fruiting russeting [42].

### 3.3. Proteome and Transcriptome Correlation Analysis

In the correlation analysis of transcriptomics and proteomics, more than 97% of proteins identified in iTRAQ proteome analysis were also covered by the transcriptomic analysis, while ~2% protein were identified only by iTRAQ, suggesting that iTRAQ and RNA-seq analysis are complementary in analyzing candidate proteins involved in specific physiology advances, such as formation of russeting in “Golden Delicious” apple. Many studies also showed that iTRAQ is more inclined to evaluate differences between low-abundance proteins and to find unknown or only suspected core protein complex members [43,44]. Previous studies have also demonstrated that the degree of correlation between the transcriptome and the proteome is generally low [45,46]. When the same or opposite expression trends of DEGs and DEPs data were analyzed in the study, the transcriptome and proteome exhibited a relatively lower correlation (r spearson = 0.36). One possible explanation for this result is that these proteins are transcribed, translated, and modified after translation, resulting in changes in proteins that are not always consistent with RNA expression. In addition, a comparative analysis of the DEGs and DEPs revealed only a small overlap between the transcriptome and proteome levels. 

### 3.4. Regulatory Factors of Lignin Biosynthesis Related Genes

In the past decade, many transcription factors of MYB family had been found to be involved in the lignin biosynthesis and their functions had been verified [47], including transcription factors that promote lignin synthesis, such as PtMYB1 [48], PtrMYB3 [49], AtMYB46, and AtMYB63 [50]. On the contrary, AtMYB4 and AtMYB32 [51], as well as EgMYB1 [52] were inhibitors of lignin biosynthesis. EMSA revealed that the AC elements contained in the promoter of related genes in the lignin anabolism pathway were consistent with the binding motif of MYB transcription factor, such as ACI (ACCTACC), ACII (ACCAACC), ACIII (ACCTAAC), PAL-box or H box (CCTACC(N)7CT), and were necessary to activate the genes like *PAL*, *4CL*, *C3H*, *CCR,* and *CAD*, etc. [34,51,52,53]. In this study, three *MYB* TF-encoding genes (MD15G1051000, MD02G1186900, and MD11G1007500) were up-regulated during the formation of apple russeting, showing that these *MYB* genes maybe upstream responsive genes in the lignin biosynthesis pathway.

However, MYB TF is not the only kind of transcription factor regulating the lignin biosynthesis pathway. For example, LIM transcription factors are also involved in lignin biosynthesis [54]. NtLIM1 in tobacco regulated the expression of *PAL*, *4CL*, and *CAD* genes, and the content of lignin decreased by about 30% after the antisense expression of *NtLIM1* [25]. The overexpression of *GhLIM1* in cotton increased the lignin content in the xylem by ~45% compared with the wild type, and played a regulatory role in the expression of key enzymes in the lignin biosynthesis pathway such as PAL, 4CL and CAD, indicating that *GhLIM1* promoted the synthesis of lignin [55]. SbLIM1 could bind to related-genes that contained PAL-box element, and the lignin content of overexpressed *Arabidopsis thaliana* plants showed a significant down-regulated trend compared with the wild type, indicating that the expression levels of related-genes involved in lignin biosynthesis were inhibited [54]. In the current study, through the functional protein association network analysis we found LIM TF had a direct interaction relationship with CAD9, and further phylogenetic analysis showed MdLIM1 was closely related to NtLIM1, indicating that it may also be involved in the lignin biosynthesis in apple russeting. The EMSA test revealed that MdLIM1 can combine to PAL-box element, indicating that it can regulate the expression of related-gene. Further luciferase reporter assays proved that MdLIM1 inhibited the expression of *MdPAL* gene through combining with PAL-box like element. 

## 4. Materials and Methods

### 4.1. Plant Materials

Fruits of “Golden Delicious” apple were collected in 2018 from the Liaoning Institute of Pomology of Xiongyue (40°10′ N, 122°07′ E; elevation 15 m), Liaoning province, China. The trees were 8 years old and their growing potential were almost identical without pests and diseases. The apples were bagged at DAF 20 according to Sun [56], 100–150 exposed fruits per tree were selected for the control group (short for CK), and 50 well fruits were bagged with light impermeable double layer (the outer layer is yellow outside and black inside, and the inner layer is red) paper bags 1202 KM-2 (KOBAYASHI (QINGDAO) CO., LTD, Qingdao, China) for the treatment group (short for T). Three trees were used as replicates for each group. Then collected samples at DAF 30, DAF 60, and DAF 150. In these three sampling periods, DAF30 and DAF60 were the two high outbreak period of apple russeting, and DAF 150 was the fruit ripening stage. The samples were marked as CK1 and T1, CK2 and T2, CK3, and T3, respectively. 

All three fruits of each sample were peeled and mixed according to Legay et. al. [20], then directly flash-frozen in liquid nitrogen and stored at −80 ℃ immediately prior to RNA and protein extraction. The experiments of RNA and protein were repeated for three biological samples from three differential stages.

### 4.2. Scanning Electron Microscope for Epidermis Structure

For SEM analysis, segments of the fruit skin were excised from russeted and non-russeted, as described previously [16]. The segments were first fixed using fixation G1102 (Servicebio, Wuhan, China) for more than 2 h and moved the segments into 1.0% osmic acid with 0.1 M phosphate buffer at room temperature 2 h for further fixation. The dehydration was performed with the different concentration ethanol, and a Critical Point Drying (Quorum K850, East Sussex, England) was used to dry the samples. Finally, placed the samples tightly on the conductive carbon film double-sided adhesive and put them into the sample table of the ion sputtering instrument (MSP-2S, IXRF, Shanghai, China) for metal spraying ~30s. Observed images with a SEM (SU8010, HITACHI, Tokyo, Japan).

### 4.3. Light Microscopy for Epidermis Cell

The segments selected method for LM was the same as described above for SEM. The segments were first fixed using FAA (G1103, Servicebio, Wuhan, China), then dehydrated and infiltrated by paraffin. After that, process tissue samples in melted paraffin in cassettes and cut 4 µm sections using Leica RM2016 microtome (Leica, Shanghai, China). The sections were dyed by SafraninO-Fast Green Staining solution (G1031, Servicebio, Wuhan, China) after deparaffinizing and hydrate to water. At last, the sections were dehydrated and mounted with resin. Sections were cut to a thickness of 3 μm and then observed images with a NIKON ECLIPSE E100 microscope (NIKON, Tokyo, Japan). The cutinized cell walls were red color and the cellulose cell walls were green color. The epidermal cell size was observed with a NIKON DS-U3 (NIKON, Tokyo, Japan), at least five biological replicates for each sample. The experiment of was repeated for three biological samples from three differential stages.

### 4.4. RNA Extraction and Sequencing

Total RNA was isolated from the fruit peel of eighteen samples of the three stages. RNA was extracted using the RNeasy Plant Mini Kit (Tiangen, Beijng, China) according to the manufacturer’s protocol. Ethanol precipitation protocol and CTAB-PBIOZOL (Bioer, Hangzhou, China) reagent was used for the purification of total RNA. Subsequently, total RNA was qualified and quantified using a Nano Drop and Agilent 2100 bioanalyzer (Thermo Fisher Scientific, Waltham, MA, USA). Limited RNA (more than 200pg, high-quality), was amplified with oligo-dT and dNTPs (KAPA HiFi HS RM, KAPA, MA, USA), incubated at 72 ℃ and immediately put back on ice, then reverse transcribed to cDNA based (Super script II reverse transcriptase, Invitrogen, CA, USA) or polyA tail. The template was switched to the 5’ end of the RNA and the full-length cDNA was generated by PCR. Agilent 2100 bioanalyzer instrument (Thermo Fisher Scientific, Waltham MA, USA) was used to determine the average molecule length of PCR product. Purified cDNA from previous steps was fragmented into small pieces with fragment buffer (5× First strand buffer, Invitrogen, CA, USA) by PCR, and the product was purified and selected by the Agencourt AMPure XP-Medium kit (Thermo Fisher Scientific, Waltham, MA, USA). cDNA was quantified by Agilent Technologies 2100 bioanalyzer. The double stranded PCR products undergo QC step was heat denatured and circularized by the splint oligo sequence. The single strand circle DNA (ssCir DNA) was formatted as the final library. The final library was quantitated in two ways in order to ensure the high quality of the sequencing data: Determined the average molecule length used the Agilent 2100 bioanalyzer instrument, quantified library used real-time quantitative PCR. Final library was amplified with phi29 (Thermo Fisher Scientific, MA. USA) to make DNA nanoball (DNB) which had more than 300 copies of one molecular DNBs were loaded into the patterned nanoarray. For each sample, the cDNA libraries were sequenced using a BGISEQ-500 System (BGI-Shenzhen, China). For each RNA sample, RIN value ≥ 6.5, A260/A280 ratio ≥ 1.8, and A260/A230 ratio ≥ 1.8. The read length of the BGISEQ-500 system was 100 bp [57].

The sequenced data were filtered and obtained clean reads, then mapped to the reference genome of *Malus × domestica* in GDDH13_v1.1 database (ftp://ftp.bioinfo.wsu.edu/species/Malus × domestica/) with Bowtie2 (http://bowtie-bio.sourceforge.net/Bowtie2/index.shtml). Then, compared clean reads to genome sequences using Bowtie2 and the gene expression levels were calculated by FPKM (fragments per kilobase million). Raw sequences have been deposited at the NCBI SRA database (SRA accession number: PRJNA560317), and Gene Expression Omnibus website (GEO, https://www.ncbi.nlm.nih.gov/geo, accession number: GSE136842). Gene annotation and functional assignments were performed based on the Nr, Swiss-prot, KEGG, and GO databases. The differentially expressed genes (DEGs) were defined by the fold change ≥2.00 and an FDR value ≤ 0.001. GO enrichment and KEGG pathway enrichment was performed to identify significantly enriched metabolic pathways for comparison with the whole genome background, according to the GO and KEGG annotation results and official classification, functional classification was conducted for the differentially expressed genes, respectively. Normally, the function of FDR ≤0.01 was considered as significant enrichment. For plant transcription factors, we used getorf to detect the ORF of unigene, and then used HMM search to compare ORF to the transcription factor protein domain (data from transcription factor), and then identified the ability of unigene according to the transcription factor family characteristics described by PlantTFDB (http://planttfdb.cbi.pku.edu.cn).

### 4.5. Protein Extraction, iTRAQ Labeling, and Mass Spectrometry Analysis

The plant materials used for the iTRAQ analysis were the same as those for RNA sequencing. Protein was extracted using a plant total protein extraction kit (PE0230, Sigma) following the manufacturer’s protocol. The quantitation of protein was determined by Bradford assay, and protein integrity was tested by 12% sodium dodecyl sulfate-polyacrylamide gel electrophoresis. The peptide labeling was performed with iTRAQ tags 113−121 (Supplementary Table S7) by iTRAQ Reagent 8-plex Kit (Applied Biosystems, Thermo Fisher Scientific, Waltham, MA, USA) according to the manufacturer’s protocol after the protein was digested by Trypsin Gold (Promega, Madison, WI, USA). The labeled peptides with different reagents were combined and desalted with a Strata X C18 column (Phenomenex) and vacuum-dried according to the manufacturer’s protocol. Then the peptides were separated on a Shimadzu LC-20AB HPLC Pump system coupled with a high pH RP column, and the eluted peptides are pooled as 20 fractions and vacuum-dried. Each fraction was resuspended and the supernatant was loaded on Thermo Scientific™ UltiMate™ 3000 UHPLC system equipped with a trap and an analytical column for testing. After that, the peptides separated from nanoHPLC were subjected into the tandem mass spectrometry Q EXACTIVE HF X (Thermo Fisher Scientific, Waltham, MA, USA) for DDA (data-dependent acquisition) detection by nano-electrospray ionization.

The raw MS/MS data is converted into MGF format by the corresponding tool, and the exported MGF files are searched by the local Mascot server against the database of *Malus* × *domestica* described above.

In addition, an automated software IQuant [58], was applied to the quantification of the labeled peptides with isobaric tags. All the proteins with a false discovery rate (FDR) less than 1% will proceed with downstream analysis including GO, COG/KOG, and Pathway. Then based on the “simple principle” (The parsimony principle), identified peptide sequences are assembled into a set of confident proteins. In order to control the rate of false-positive at protein level, a protein FDR at 1%, which is based on Picked protein FDR strategy [59], will also be estimated after protein inference (Protein-level FDR ≤ 0.01). The differentially expressed proteins (DEPs) were defined by the fold change ≥ 1.20, and Q-value < 0.05. And the mass spectrometry proteomics data have been deposited to the ProteomeXchange Consortium via the PRIDE [60] partner repository with the dataset identifier PXD014960.

### 4.6. Protein and RNA Correlation Analysis

To investigate the concordance between transcriptome and proteome levels during the three stages, a correlation analysis was performed from the expression and functional enrichment aspects. The expression ratios based on log2 fold change of DEGs and DEPs were defined as parameter of correlation analysis. Significant pathway enrichment was examined using the Pearson test and the *p*-value < 0.05. Main parameters table of correlation analysis were listed in the Table S9. Considering that bagging can also affect fruit color, phenolic compounds, anthocyanin biosynthetic, and regulation pathways, so we focus our research mainly on alterations in the exocarp cell wall in order to avoid interference factors.

### 4.7. Quantitative Real-time PCR Validation

The twenty genes that conjoint by the transcriptome and proteome were selected for validating gene expression by quantitative real-time PCR (qRT-PCR). All the primers were designed by online Primer-Blast software (https://www.ncbi.nlm.nih.gov/tools/primer blast/index.cgi?LINK_LOC= BlastHome) and synthesized by Genewiz Company (China). A total of 1.0 μg of RNA was used for synthesizing cDNA using a PrimeScript RT regent Kit with gDNA Eraser (TaKaRa, Japan) according to the manufacturer’s protocol. qRT-PCR was performed on the CFX96 ^TM^ Real-Time System (Bio-Rad Laboratories, USA) using the following cycling conditions: 95 ℃ for 3 min, and 40 cycles of 95 °C for 5 s, 58 °C for 30 s, 72 °C for 30 s, followed by a melting curve analysis. Each reaction mix contained 2.0 μL previously diluted cDNA (1:5), 12.5 μL TB Green Premix DimerEraser (Perfect Real Time) (TaKaRa, Japan), 7.5 mM of each primer, for filling a final volume of 25 μL using 9.0 μL RNase-free water. At least three biological replicates were performed for PCR amplification, and all of the primers are listed in Table S10. And the relative gene expression values were calculated using the 2^−ΔΔCt^ method, we used *Actin* (Forward primer: 5′-TGACCGAATGAGCAAGGAAATTACT-3′ and reverse primer: 5′-TACTCAGCTTTGCAATCCACATC-3′) and *18S rRNA* (Forward primer: 5′-GACGGATCGCACAGCCATC-3′ and reverse primer: 5′-GGATTGGGTAATTTGCGCGC-3′) as the housekeeping genes.

### 4.8. Functional Association Network

A lignin-related functional association network was built using the online software STRING (http://stringdb.org) from the TAIR code obtained from related genes of lignin biosynthesis (Supplementary Table S6). The confidence level of minimum required interaction score parameters was set at 0.3.

### 4.9. Phylogenetic Analysis

The sequences of LIM protein-coding gene from GDDH13 genome (https://iris.angers.inra.fr/gddh13/) and PLAZA database (https://bioinformatics.psb.ugent.be/plaza/versions/plaza_v3_dicots/), then the sequences was converted into amino acid sequences by SMS2 (http://www.detaibio.com/sms2/trans_map.html). Other LIM proteins of fourteen plants (*A. thaliana* (http://www.arabiodpsis.org), *G*. *hirsutum*. (http://mascotton.njau.edu.cn), *N. tabacum*, *C. sinensis* Osbeck, *E. globulus*, *F. ananassa*, *G. max*, *G. raimondii, M. truncatula, P. patens, S. bicolor, S. tuberosum, V. vinifera,* and *Z. mays* (https://phytozome.jgi.doe.gov/pz/portal.html). All of the amino acid sequences were used for a protein family clustering analysis. Used Clustal W [61] to perform the sequence alignments, and used MEGA 7.0 software [62] to construct phylogenetic trees with the neighbor-joining (NJ) method [63]. The bootstrap analysis was set to 1000 replicates. The amino acid sequences were in Table S8. 

### 4.10. Electrophoretic Mobility Shift Assay

The coding region of MdLIM1 (MD15G1068200) was cloned into the pGEX4T-1 vector at KpnI—SalI. The two primers were (5′-GGATCCATGGCATTTGCAGGAAC-3′ and 5′-GTCGACTCATATTTCAGCAGCAACTTC-3′). The recombinant vector was transformed into BL21 (DE3) for expression. The electrophoretic mobility shift assay (EMSA) was performed using the Light Shift Chemiluminescent EMSA Kit (#89880; Thermo Scientific), according to the manufacturer’s protocol. The unlabeled probes, biotin-labeled probes, and unlabeled mutant probes at the 3′ end were synthesized by Genewiz Co., Ltd. (Supplementary Table S10). The probe sequence came from PAL-box element (CCACTTGAGTAC) of *Arprxc2* of *Armorocia Rusticana*. The samples of protein-DNA complexes were separated on 6.5% acrylamide gels, and transferred to a nylon membrane, and signals were captured using ChemiDoc MP Imaging System (BIO-RAD).

### 4.11. Luciferase Reporter Assays in Nicotiana Benthamiana Leaves

The coding sequence of MdLIM1 (MD15G1068200) was cloned into the pRI101-AN vector at the BamHI—SalI sites, generated the reporter construct pRI101-AN: MdLIM1. The two primers (5′-GTCGACTCCTATTCCGAAGAAGGC-3′ and 5′-GGATCCGTTAATTACGAGAAACT GAAAAC-3′) were used for amplifying the promoter region (~1231 bp upstream of the start codon ATG) of *MdPAL* (MD04G1096200) (Supplementary Figure S7), and it was cloned into pGreenII 0800-LUC vector(came from The New Zealand Institute for Plant & Food Research Limited) at SalI—BamHI sites, resulting in the reporter construct pMdPAL:*LUC*. Two reporter constructs were transformed into *Agrobacterium tumefaciens* strain GV3101. The reporter construct pMdPAL:*LUC* was used as control, and the reporter construct pMdPAL:*LUC* and pRI101-AN:MdLIM1 co-injection were used as treatment. Infiltrating bacterial suspensions were infiltrated into young leaves of the 2-month-old *N. benthamiana* plants by a needleless syringe. After infiltration, plants were grown first under dark for 12 h and then with 16 h light/8 h dark cycle for 3 days at 25 °C. The leaves were sprayed with 100 mM luciferin and maintained under dark condition for 2 min. The LUC images were captured in a low-light cooled CCD imaging apparatus (Tanon 5200Multi, China). The experiments were repeated independently at least three times with similar results.

## 5. Conclusions

In this study, the DEGs and DEPs were identified during fruit russeting formation of “Golden Delicious” apple, and a correlation analysis based on transcriptomics and proteomics was carried out. Related-genes involved in phenylpropanoid and lignin biosynthesis pathway were significantly changed at three developmental stages. Furthermore, several transcription factors that regulated lignin biosynthesis correlated with apple fruit russeting formation. We also speculate that one apple LIM transcription factor will provide a valuable perspective on the molecular mechanisms controlling apple fruit russeting formation.

## Figures and Tables

**Table 1:** Genes involved in phenylpropanoid biosynthesis during the formation of fruit russeting.

Gene ID	Gene Length (bp)	Log2(T/CK)	Description	Ko Number
MD01G1042500	2011	−2.41	CAD, cinnamyl-alcohol dehydrogenase [EC:1.1.1.195]	K00083
MD01G1042800	1779	−2.66	CAD, cinnamyl-alcohol dehydrogenase [EC:1.1.1.195]	K00083
MD02G1161000	2472	−2.02	PDA, phospholipid: diacylglycerol acyltransferase [EC:2.3.1.158]	K00679
MD03G1126700	5941	−1.39	PDC, pyruvate decarboxylase [EC:4.1.1.1]	K01568
MD04G1096200	3458	−1.81	PAL, phenylalanine ammonia-lyase [EC:4.3.1.24]	K10775
MD06G1194000	1697	−2.24	UBR4, E3 ubiquitin-protein ligase [EC:2.3.2.27]	K10691
MD11G1153800	3440	−1.56	PDA, phospholipid: diacylglycerol acyltransferase [EC:2.3.1.158]	K00679
MD11G1210200	1645	−2.72	UBR4, E3 ubiquitin-protein ligase UBR4 [EC:2.3.2.27]	K10691
MD11G1214800	2056	−1.86	PDA, phospholipid: diacylglycerol acyltransferase [EC:2.3.1.158]	K00679
MD12G1252500	2161	1.32	PDA, phospholipid: diacylglycerol acyltransferase [EC:2.3.1.158]	K00679
MD17G1157600	2990	−2.25	HCT, shikimate O-hydroxycinnamoyl transferase [EC:2.3.1.133]	K13065

**Table 2:** Genes involved in cutin, suberine, and wax biosynthesis during the formation of fruit russeting.

Gene ID	Gene Length (bp)	Log2(T/CK)	Log2(RAE/WAE)	Description	Ko Number
MD03G1073600	1904	−2.79	3.97	CYP86A1, long-chain fatty acid omega-monooxygenase [EC:1.14.14.80]	K15401
MD08G1058900	1808	1.67	-	HHT1, omega-hydroxypalmitate O-feruloyl transferase [EC:2.3.1.188]	K15400
MD09G1007600	1797	−2.23	-	HHT1, omega-hydroxypalmitate O-feruloyl transferase [EC:2.3.1.188]	K15400
MD13G1004700	2106	−2.84	4.75	CYP86B1, fatty acid omega-hydroxylase [EC:1.14.-.-]	K15402
MD17G1011500	1790	−2.27	-	HHT1, omega-hydroxypalmitate O-feruloyl transferase [EC:2.3.1.188]	K15400

Note: The values of Log2(RAE/WAE) come from reference PMID: 25786603.

**Table 3:** 20 differentially expressed proteins (DEPs) of comparative groups during the formation of fruit russeting.

Protein ID	Mass (kD)	Mean Ratio (T/CK)	Description	Ko ID
MD01G1205400	33.79	0.60	At4g11410, WW domain-containing oxidoreductase	K19329
MD02G1009300	53.71	0.67	At2g24270, glyceraldehyde-3-phosphate dehydrogenase (NADP+) [EC:1.2.1.9]	K00131
MD02G1157800	35.27	0.58	-, photosystem II oxygen-evolving enhancer protein 1	K02716
MD05G1122100	42.35	0.65	At1g68010, hydroxypyruvate reductase 1 [EC:1.1.1.29]	K15893
MD05G1209300	40.59	0.63	At3g14420, (S)-2-hydroxy-acid oxidase [EC:1.1.3.15]	K11517
MD08G1007200	16.92	0.67	-, plastocyanin	K02638
MD08G1168600	48.75	0.60	At5g08640, flavonol synthase [EC:1.14.20.6]	K05278
MD09G1050500	49.97	0.74	At5g39830, HtrA serine peptidase 2 [EC:3.4.21.108]	K08669
MD09G1252100	20.72	0.58	-, ribulose-bisphosphate carboxylase [EC:4.1.1.39]	K01602
MD09G1262800	41.17	0.65	At1g66430, fructokinase [EC:2.7.1.4]	K00847
MD10G1011900	19.93	0.59	At4g03520, thioredoxin 1	K03671
MD10G1195700	52.49	0.55	At3g14420, (S)-2-hydroxy-acid oxidase [EC:1.1.3.15]	K11517
MD14G1219000	41.45	0.69	-, chitinase domain-containing protein 1	K17525
MD15G1006300	16.86	0.72	-, plastocyanin	K02638
MD15G1222600	23.68	0.64	At3g26060, peroxiredoxin Q/BCP [EC:1.11.1.15]	K03564
MD15G1272500	35.24	0.53	-, photosystem II oxygen-evolving enhancer	K02716
MD15G1307200	56.88	0.53	At4g37930, glycine hydroxymethyl transferase	K00600
MD15G1421500	27.39	0.69	-, exocyst complex component 2	-
MD17G1020900	32.92	0.67	At4g24770, nucleolin	K11294
MD17G1132900	28.77	0.56	-, photosystem II oxygen-evolving enhancer	K02717

**Table 4:** Correlation analysis of differentially expressed proteins (DEPs) and differentially expressed genes (DEGs) related to lignin biosynthesis.

ID	Protein	Gene	Description	Ko ID
	T/CK	Log2(T/CK)		
MD00G1116600	1.72	−1.77	UBR4, E3 ubiquitin-protein ligase	K00679
MD03G1059200	0.67	1.31	PRX, Peroxidase	K00430
MD08G1009200	0.84	−1.21	CAD, Cinnamyl-alcohol dehydrogenase	K00083
MD17G1092400	1.42	−1.85	PRX, Peroxidase	K00430
MD17G1225100	1.00	1.12	HCT, Shikimate O-hydroxycinnamoyl transferase	K13065

## Supplementary Materials

Supplementary materials can be found at https://www.mdpi.com/1422-0067/20/18/4462/s1.

## Abbreviations

DAF	days after flowering
SEM	scanning electron microscopy
LM	light microscope
PAL	phenylalanine ammonia-lyase
CCR	cinnamyl CoA reductase
CAD	cinnamyl-alcohol dehydrogenase
POD	peroxidase
PRX	peroxidase
C4H	cinnamate hydroxylase
C3H	coumaric acid 3-hydroxylase
4CL	4-coumaric acid- CoA ligase
PDC	pyruvate decarboxylase
HCT	shikimate O-hydroxycinnamoyl transferase
PDA	phospholipid: diacylglycerol acyltransferase
DEGs	differentially expressed genes
DEPs	differentially expressed proteins
qPCR	Quantitative real-time PCR
iTRAQ	isobaric tags for relative and absolute quantitation
RNA-Seq	RNA sequencing
NR	NCBI non-redundant protein sequences
KEGG	Kyoto Encyclopedia of Genes and Genomes
Ko	KEGG ortholog
GO	Gene ontology
TF	transcription factor
HPLC	high-performance liquid chromatography
MS/MS	tandem mass spectrometry
NJ	Neighbor-joining
