# Supplementary material for: An Integrated Transcriptome and Proteome Analysis Reveals New Insights into Russeting of Bagging and Non-Bagging “Golden Delicious” Apple
Source: Int J Mol Sci. 2019 Sep 10;20(18):4462. doi: 10.3390/ijms20184462 (PMC6769969; doi:10.3390/ijms20184462)
Supplement: Supplementary file 1 [file ijms-20-04462-s001.zip › ijms-566910-SI/Supplementary files/Supplementary Figure.docx]

*
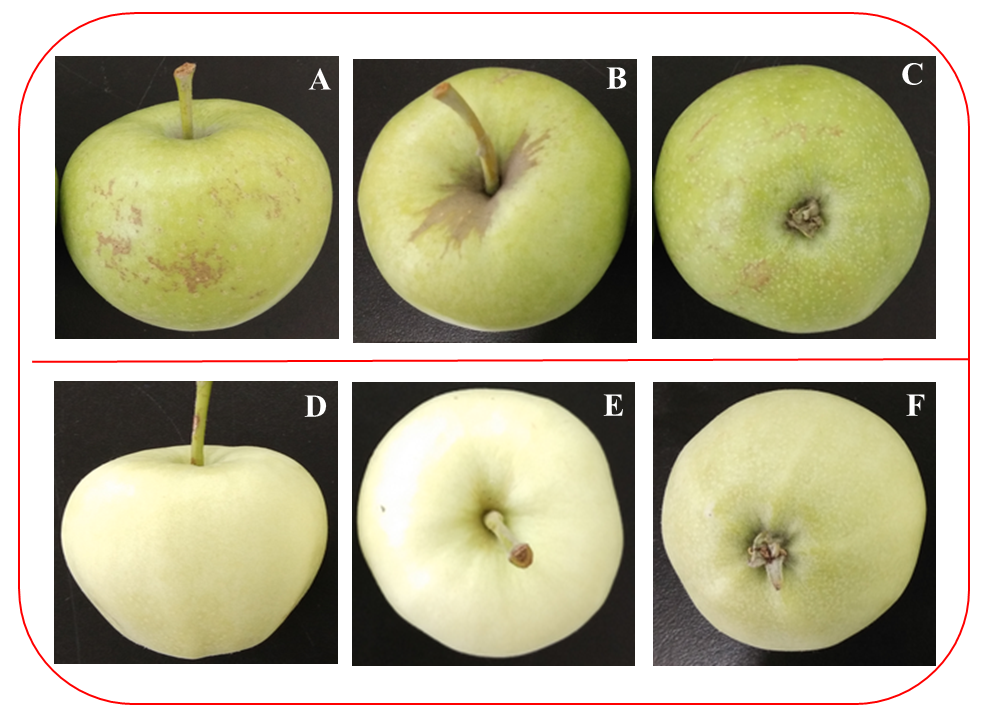
*

**Supplementary** **Figure 1.** The position of russeting in ‘Golden Delicious’ apple at DAF60: (A/D) stem hollow; (B/E) stalk; and (C/F) top of apple body. A, B, C belong to un-bagged fruits, shaort for CK group, and D, E, F belong to bagging fruits, shaort for T group.


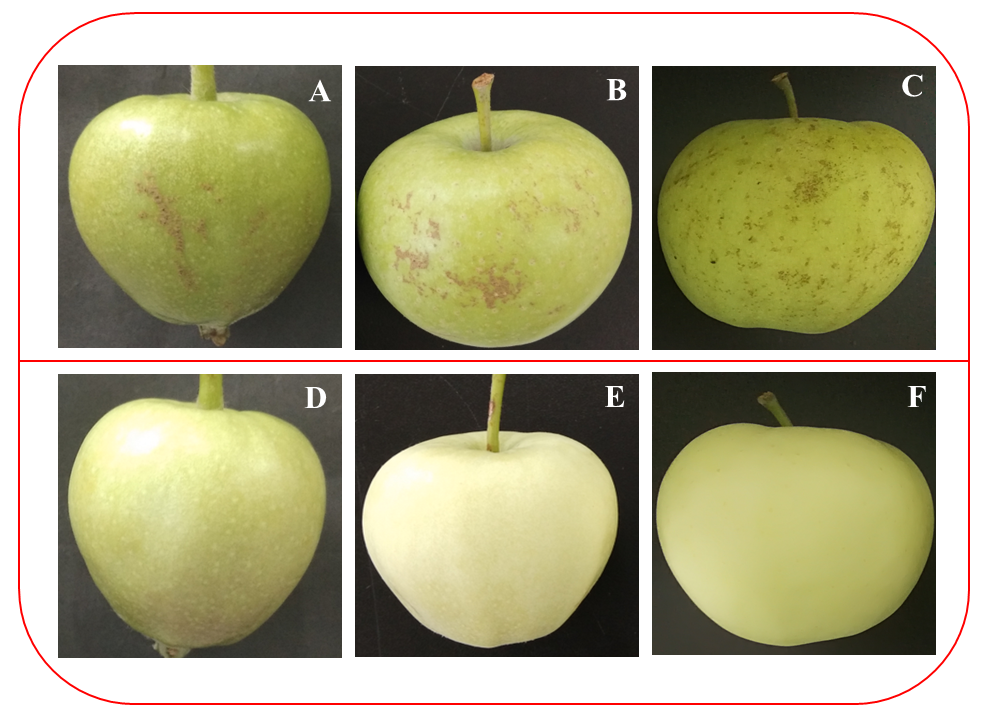


**Supplementary** **Figure 2.** The formation process of russeting in ‘Golden Delicious’: (A/D) DAF30; (B/E) DAF60; and (C/F) DAF150. A, B, C belong to CK group, and D, E, F belong to T group.

**Supplementary** **Figure 3.** Thickness of the wax layer and size of the epidermal cells: (A): thickness of the wax layer at DAF30, DAF60, DAF150 of T and CK, respectively; and (B) size of the epidermal cells at DAF30, DAF60, DAF150 of T and CK, respectively. The different small letters within the same column means significant difference at P<0.05.


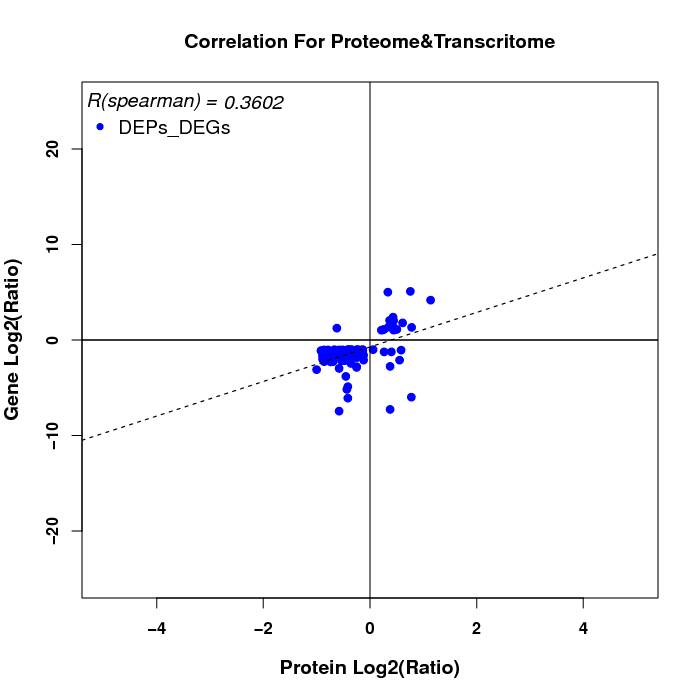


**Supplementary** **Figure 4.** The association diagram of DEGs and DEPs. R represents spearman correlation. The X-coordinate is the expression level of protein, and the Y-coordinate is the expression level of gene.


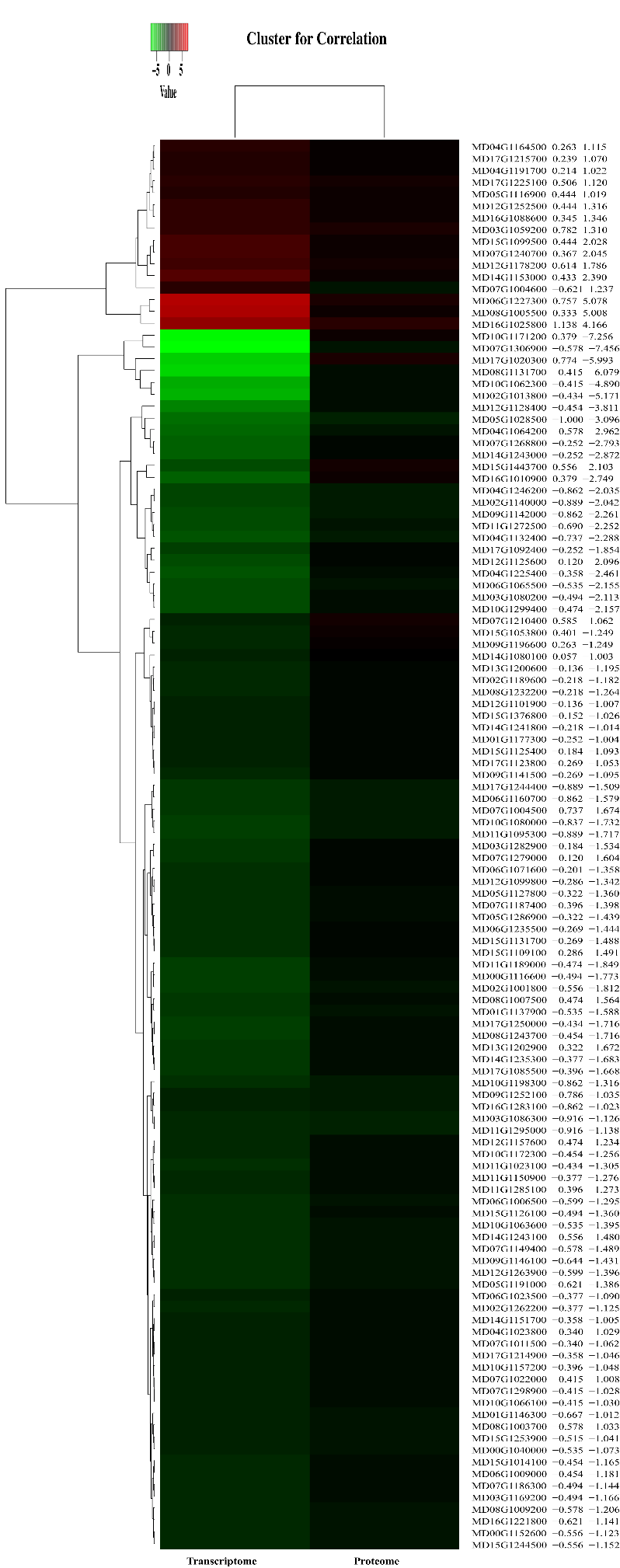


**Supplementary** **Figure 5.** Cluster analysis of quantitative DEGs and DEGs.


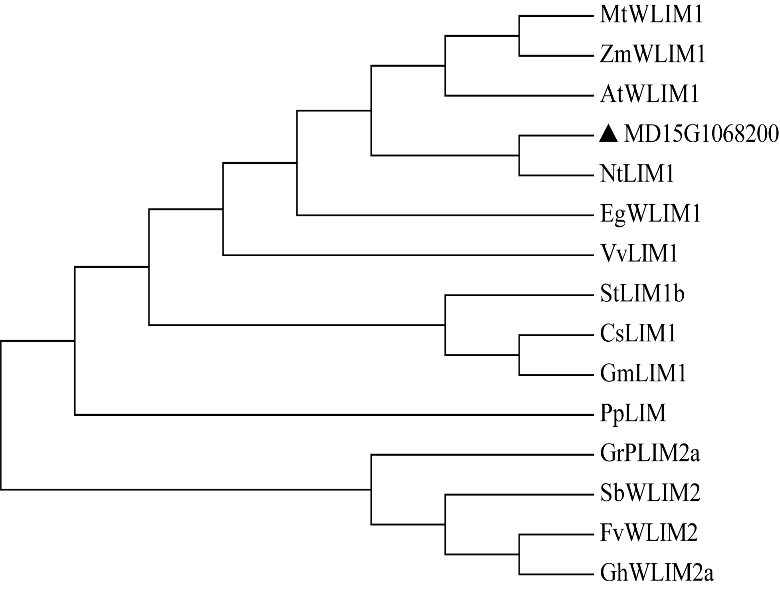


**Supplementary Figure 6. Phylogenetic analysis of LIM TFs.**

TCCTATTCCGAAGAAGGCAATGGTTTTCTTACTTTGTACTCCAAATTGACATCAACAAATGTTCA

TCCATGTCACCATTTATGTGGTTGCCCTCACCATTATAGATAACCAACACAGCCACGTATATTTA

TCTCATTAGATATAAGAGCAACTCCAGCGTATCAAATGCCCCTTAGGGTAACTTACTATTGAATC

CCCCAAGTGAACAATAAATGCCTTTAATGAACAGTAGCTGCCGTTGGATTAAAAAAATTTTGAAA

TCCAACCCTCCAGATTGTGCCACGTGGCACACGATAACATATCTGAATTTTTTAATGTGAATTTT

TTTTTTTAAATTTAAAGGCGAATAACTTGGACCGTTGATCTCAAATCCAATAGCTGATAAAAAAA

GTAACCGTTGAAATCCAATGGACGAGAACGTGCCAGCCCACCAACGGTAACATTTCAGCTGTCTG

CATCGCGGGCCCCATTGACTGAAAAGTTGTAGGTGACGCGCCCCCACGCGCACGTATGCGACACG

CGCCTGACGCAAATTTTTTTATACGTGGCTGACCATGCATCATTTGACCTTAGGCTCTCGGGCCA

CTGATTCGAGCCGGGCCTTTCACTCGGGCCCCCCTCAACCCAATTACCTGCATGGGCTGGAGCAA

GGAGCTGGGGCTATTGGGTCAAAAAAATTGCTTGTCCATCGGGCTATTGATCCCGGTGGAGTTGC

TCTAAGTGCCCCTCCTTATTCCATACACATACAAGTTGACCATAAATATTCATTTTATATATAGT

TTTTCCTTTTTCACACTAACAACAATCCCCACCAAACACTTTCATCTCCTCCTACCCCAGAAACC

CAAAGCAACCAACAAAGCATACGGTCGTCCAATCGTCCACCTCAGTAGCCACGTTGCAAGAACCT

PAL-box

CTCCACCGATGGATCATGCCACCATCGCACAGCTACATTGAATTTTCAACCAACCCGCGTGTGGC

TCTCTGCCACCCTTATGCTCACCTACCAAGCCACTTCCCAAATATGAGTCGTTTCAAAACCCCAT

TATA box

CCTTTCCTTCCACTTACCCCCTCCTCTCTATTTAAACCTCCTCCTCCCTCCCTCCACTCCTCAAA

AACCAACACTCCACCAACGGTCCACCACCACCTTTTTATCTTTTCACCACTTTCTTTAAACACCT

TCCTCGCTTCTACTTCCCTCGGTTTTTAGAGTTTTTGAGTTTTCAGTTTCTCGTAATTAAC

**Supplementary** **Figure 7.** Promoter analysis of MdPAL
